# Automated framework for multi-domain social media text analysis for business strategy employing multilayer perceptron with Word2Vec features and LIME XAI

**DOI:** 10.1371/journal.pone.0336240

**Published:** 2025-11-13

**Authors:** Amira Turki

**Affiliations:** College of Business Administration, University of Business and Technology, Jeddah, Saudi Arabia; Chunghwa Telecom Co. Ltd., TAIWAN

## Abstract

Sentiment analysis is a pivotal domain in Natural Language Processing (NLP), particularly for understanding opinions expressed in sequential and textual data with the usage of machine learning. It involves identifying and categorizing emotions expressed in textual reviews and messages. Social media platforms such as Twitter, Facebook, and Instagram generate extensive datasets rich in sentiments, making their analysis crucial for monitoring public opinion and informing business strategy. By uncovering customer satisfaction levels, product feedback, and service-related concerns, sentiment analysis helps organizations refine marketing efforts, optimize product features, and improve service delivery. Traditional machine learning techniques struggle to process large datasets and yield accurate results efficiently. To address this, we propose an effective multi-layer perceptron deep network with word embedding features, called MultiSentiNet, for sentiment analysis on Twitter datasets. The proposed model’s performance is evaluated against conventional machine learning classifiers and state-of-the-art deep learning classifiers, indicating superior accuracy with three different datasets. The significance of the proposed model is further tested on three diverse datasets (women’s e-commerce, US airline sentiments, and hate text-speech detection) that demonstrate that the proposed framework outperforms other classifiers in terms of accuracy, recall, precision, and F1 score. The performance of the proposed model is compared with previously published research works. Furthermore, the interpretability and analysis of MultiSentiNet results are explained using the LIME XAI technique, providing deeper insights into the model’s predictions and practical value in strategic business decision-making.

## 1 Introduction

Natural language processing (NLP) has witnessed great development, particularly with the rise of social media platforms and the application of artificial intelligence approaches. In essence, text analysis and text mining from social media platforms carry valuable insights [38]. Information from such platforms is extracted from raw data using text mining, which is a distinguished research area within the data mining domain. Text mining is an undeniable reality to retrieve and monetize vital information from a massive amount of daily generated data worldwide with an estimated volume of 2.5 quintillion bytes [1,2]. Text mining has a newly emerged and prominent research area called text classification, particularly with the widespread adoption of X.com (Twitter), Facebook, and WhatsApp as social media platforms. These platforms provide a space for people to express their opinions, which can serve as valuable guidance for shaping the strategies of several businesses. For instance, user sentiments related to a particular product or company can be revealed by scrutinizing the related tweets helping in user service improvement and product acceptance enhancement strategies formulation. The wide social platforms’ adaptation and usage result in the generation of copious data containing a diverse range of potentially valuable information.

The rapid advancements in internet technology, coupled with the ongoing evolution of web version 2.0, have generated a significant daily data volume. The proliferation and diversity of social media have heightened the connected users, fundamentally transforming conventional notions of personalization, socialization, and networking. In quarter 4 of 2020, Facebook alone boasted approximately 1.8 billion active daily users [3]. This figure does not even include the substantial user base of Meta’s associated amenities like Messenger, WhatsApp, and Instagram, each of which boasts a monthly active user base in billions [4,39]. Likewise, third-party analyses indicate that other major social media platforms like Apple, Tencent WeChat, and Google YouTube (now no longer exclusive) have all joined the coveted club of platforms with a billion active users monthly. Additionally, a whopping 3/4 of the internet user base regularly interacts with any one of the social media platforms [5]. This surge in accessibility has presented both new opportunities and challenges. It has encouraged users not only to share their perspectives, emotions, and opinions but also to consume a diverse range of services [6,7]. Among the swiftly fostering and influential social networks, X (Twitter) stands out. Here, users can interact by writing tweets, reposting other people’s tweets, and commenting on other’s tweets in concise text messages, which serve as a platform for expressing personal sentiments, opinions, and views about various subjects. Sentiment-rich tweets play a pivotal part in numerous domains, including social media marketing, academia, and the dissemination of news during election campaigns [8].

Sentiment analysis is a method of classifying and defining the subjective text’s polarity at each and every level [9]. It is applicable in various real-world domains like infotainment, sports, business, politics, healthcare, and much more. For example, it can assist companies in monitoring customer perceptions of their products and help customers make informed decisions based on public opinion [10]. Despite its wide-ranging applications, sentiment analysis faces diverse challenges associated with Natural language processing (NLP). Ongoing sentiment analysis research is still undergoing theoretical and technical complexities inhibiting sentiment detection accuracy [11]. Hussein *et al*. [12], explored the encountered problems and their impact on the accuracy of sentiment analysis. The investigational findings confirmed a significant challenge of attaining high accuracy in conducting sentiment analysis. The study also highlighted that accuracy is influenced by various factors, including handling sarcasm, negation, abbreviations, domain-specific context, and the presence of bi-polar words, among others.

The sentiments on X (Twitter) tweets are analyzed through machine learning (ML), and traditional approaches. In the ML approach, sentiments are classified using learning models. The rule-based method relies on sentiment lexicons derived from corpora, publicly accessible sentiment lexicons, or lexical dictionaries to extract sentiments. The hybrid approach combines elements of both the rule based and ML approaches. Accordingly, deep learning (DL) methods, unified into numerous research studies, have demonstrated their significance in speech recognition, computer vision, and sentiment analysis domains. This architectural approach in deep learning resembles human brain functioning. It is composed of multiple layers to process the information and then pass it on to the subsequent layers. Each layer refines the results iteratively during its cycle. At every iteration, the desired results are compared to the original labels to fine-tune the feature weights to achieve higher accuracy accordingly. Various types of deep learning networks are presented, which include recurrent neural networks, multilayer perceptrons, deep neural network (DNN) deep restricted Boltzmann machines (RBMs), convolutional neural network (CNN), and long short-term memory networks (LSTM) [35]. But DNNs and MLPs are the prominently deployed layered architecture.

The study on sentiment analysis has gained prominence across various domains including the airline industry, E-commerce, and the identification of hate speech in text. The objective is the enhanced revenue generation and formulation of new techniques by refining services from a future prospective. Customer feedback through social networks is a driving force for such enhancements, as valuable insight is derived through customer reviews and concerns. Analyzing such reviews relies on the expressions conveyed within them. Given the high volume of these reviews, a considerable workforce of experts would be needed for analysis and categorization. Consequently, numerous classifiers based on deep learning are suggested to alleviate the human effort required for review classification. However, there is still room for improvement in order to elevate accuracy.

This paper recommends the utilization of an MLP classifier for this purpose with the objective of assessing the performance of well-known ML and deep learning classifiers across a variety of datasets spanning different domains. The key contributions of this research are as follows

- This research introduces a novel approach for conducting sentiment analysis on Tweets, employing an MLP model. Two discrete feature mining techniques, namely word2vec and term-frequency and inverse document frequency(TF-IDF) are employed to assess the effectiveness in conjunction with the proposed approach.
- The recommended technique is rigorously evaluated and compared with a suite of ensemble classifiers, including random-forest (RF), support vector classifier (SVC), logistic regression (LR), and stochastic gradient descent (SGD), as well as a voting classifier (VC) consisting SGD and LR.
- The efficacy of the suggested approach is evaluated involving datasets from multiple domains. This comparative analysis serves to provide valuable insights into the performance and applicability of this recommended approach in relation to existing top-notch models.

The remaining paper is structured in this way: Sect 2 overviews the research precisely pertinent to the present study. Sect 3 describes the proposed approach and datasets used for experiments. Sect 4 presents the findings, analysis, and discussions while Sect 5 covers the conclusion and offers suggestions for future work.

## 2 Related work

A broad scope for sentiment analysis exists in the text classification domain, and numerous researchers have investigated the sentiment analysis process. Umer *et al*. [13] investigated the influence of CNN and FastText embedding on text classification. To this end, they trained the classification model, Fast Text, as a leading word embedding generation model on publicly available datasets [40]. This included six benchmark datasets such as Amazon Full and Polarity, Ag News, Yelp Full, and Polarity, and Yahoo Question Answer. Additionally, the recommended prototype underwent testing on the normal dataset of Twitter US airlines. The study results in an accuracy of 86% with FastText embedding. For the automated classification of tweets, a voting classifier was introduced by Rustam *et al*. [14]. The VC relies on LR and SGDC, employing a soft voting mechanism to produce the final prediction. The study demonstrated that the proposed voting classifier achieved a classification accuracy of 79.1% when employing TF-IDF features.

Rane and Kumar [15] focused on the sentiment classification of tweets related to US airlines. For phrase-level analysis, they implemented the deep learning-based doc2Vec features. They utilized seven different ML models for classification. The outcomes revealed that an accuracy of 84.5% was attained by the AdaBoost model. In a separate study, Lakshmanarao *et al*. [16] developed a deep learning fusion system [41] for classifying tweets concerning six US airlines. They employed LSTM, CNN, and an ensemble of CNN-LSTM models based on deep learning. The findings indicated that the proposed deep ensemble model CNN-LSTM achieved an accuracy score of 93%.

Mahmud *et al*. [17] introduced an ML-driven approach for sentiment classification of women’s clothing reviews on Bangladesh e-commerce platforms. This study incorporated five ML models in conjunction with a deep learning model, LSTM. The results highlight that the ML model RF demonstrated superior performance, achieving an accuracy score of 96.51%. In another study, Xiaoxin Lin [18] suggested an ML-based system for the efficient classification of e-commerce clothing reviews. The author employed five widely recognized ML algorithms, including SVM, XGBoost, RF, LR, and LightGBM. These algorithms were employed to find relationships between review features and product recommendations through NLP. The results of the study indicated that LightGBM outpaced the other learning models, attaining an impressive accuracy score of 98%.

Balakrishnan *et al*. [19] introduced a deep learning-based ensemble approach for classifying clothing reviews. They leveraged various word embedding techniques like bidirectional encoder representation using a transformer (BERT), FastText, and word2vec. The experiments were conducted on both the original and augmented datasets, evaluating all models in two setups: 5-class versus 3-class classification. The results demonstrated that the ensemble model CNN-RNN-BiLSTM achieved the highest 96% accuracy on the augmented dataset using 3 classes. In a separate study, Zubrinic *et al*. [20] proposed an ML-based system for classifying women’s e-commerce clothing reviews. They employed ML models including Naive Bayes (NB), SVM, MLP, J48, and logistic classifier. The findings revealed that the SVM model achieved an accuracy of 84.5%.

Identifying hate speech online poses a significant challenge. Researchers worldwide have made substantial contributions toward detecting such content. Patil and Pattewar [21] introduced an ML-based system for hate speech detection automatically. They utilized the Kaggle TwitterHate dataset, consisting of 31,962 tweets categorized as either hate or non-hate, to evaluate their proposed method. The suggested approach underwent testing using commonly employed ML classifiers with a multi-model technique. With the use of TF-IDF features, the detection results achieved an accuracy of 96.29%. For automated hate speech detection on online platforms, Satpute *et al*. [22] presented an ML-based system. They employed RF and NB for classifying a dataset collected from Twitter using the Twitter API. This study’s results demonstrated that the RF ML model achieved an accuracy score of 81.21% for hate speech detection. In Table 1, summary of all previous work is discussed.

**Table 1:** Accuracy comparison of proposed appraoch with previous research works.

Ref	Classifier	Dataset	Achieved accuracy
[13]	RF, ETC, LR, GBM, CNN, SGD	US airlines	86% by CNN
[14]	ADA, Calibrated, DT, ETC, SGDC, SVC, VC(LR+SGDC)	US airlines	79.1% by VC using TF-IDF features
[15]	DT, RF, SVM, KNN, LR, GNB and ADA	US airlines	84.5% by ADA
[16]	CNN, LSTM, CNN-LSTM	US airlines	93% CNN-LSTM
[19]	CNN, Bi-LSTM, RNN, CNN-RNN-Bi-LSTM	Women’s e-commerce clothing reviews	83.65% using CNN-RNN-Bi-LSTM
[20]	NB, SVM, MLP, J48, Logistic	Women’s e-commerce clothing reviews	82.50 SMO
[21]	LR, XGB, ETC, ADA, SVM, Majority voting	TwitterHate dataset	96.29% Majority voting on TF-IDF features
Proposed	**MultiSentiNet**	**Multi-Domain**	**Hate Speech 97.52% US Airline 94.75% Women E-commerce 84.60%**

## 3 Materials and methods

This section provides a comprehensive overview of the MultiSentiNet framework. Additionally, it delves into the specifics of experimentally deployed datasets. Furthermore, a concise explanation of the chosen ML and DL classifiers is included.

### 3.1 Datasets utilized in this study

This study conducts sentiment analysis using 3 datasets. The rationale behind utilizing several datasets is to assess the effectiveness of the projected model across diverse data sources. Furthermore, the reason for selecting these three diverse and real-world datasets—women’s e-commerce, US airline sentiments, and hate text-speech detection—is to evaluate the robustness of the proposed MultiSentiNet model across varying domains, sentiment contexts, and class distributions. These datasets were chosen to represent key business, customer service, and social issues, each posing unique challenges, including imbalance, domain-specific jargon, and contextual polarity. Each dataset comprises varying numbers of Tweets along with their respective classes. Table 2 breakdowns each dataset comprehensively, including total record count and class distribution. Given that each dataset encompasses varying quantities and types of attributes, it is advisable to first elucidate this study deployed attributes’ specifications prior to embarking on data visualization. Table 3 provides a comprehensive overview of all the utilized datasets.

**Table 2:** Datasets details.

Dataset name	Rows count	Class
Dataset 1: US-airline-sentiment (D1) [32]	14640	3
Dataset 2: Women-E-commerce-clothing-reviews (D2) [31]	23486	5
Dataset 3: Hate-text-speech-detection (D3) [44]	29530	2

**Table 3:** Datasets description.

Dataset 1 - Twitter airline sentiment	
Feature	Description
Sentiment confidence	Confidence level is represented numerically to assign tweets to one of the three classes.
Negative reason	Assigning the reasoning for the negative classification of the tweet
Negative reason confidence	This determines the level of confidence behind a negative tweet for the negative reason.
Airline	Name of the Airline operator.
Retweet count	The retweet stats for a tweet.
Text	User posted original tweet.
Airline sentiment	Tweets Labels (negative, neutral, positive).
**Dataset 2 - Women-E-commerce-clothing-reviews**	
Feature	Description
Clothing-ID	Individual product ID.
Customer-Age	Reviewer-Age
Caption	Reviewer-Caption
Review-Text	User posted original text
Rating	Reviewer assigned product rating.
Recommended IND	Recommendation or refusal for the product being reviewed
Positive Feedback-Count	Positive-feedback numbers on review.
Department Name	Product department-Name.
Division Name	Name of product division.
Class-Name	Product-type
**Dataset 3 - Hate-text-speech-detection**	
Label	Tweet label class
Sentiment-Text	User posted original-text

### 3.2 Data visualization

Visualization is crucial to comprehend a dataset, aiding in the identification of significant patterns before implementing a classification model. In Dataset 1, Twitter data pertaining to 6 airline operators including US Airways, American Airlines, United Airlines, Southwest Airlines, Delta Airlines, and Virgin America Airlines is collected. Each airline has a distinct number of Tweets. Notably, United Airlines boasts the highest share, constituting approximately 26% of the dataset. Dataset 1 further encompasses ten distinct negative reasons associated with each airline. The counts for these reasons vary significantly. It is observed that the most prominent concern voiced by customers pertains to customer service issues [26].

Likewise, 20 distinct garment classes make up tweets containing the dataset, with Pants, Sweaters, Dresses, Knits, and Blouses being the most prevalent categories. Meanwhile, Dataset 3 consists of Tweets featuring both adversarial and supportive reviews, with the objective being to classify them as either hate or non-hate expressing sentiment.

### 3.3 Data preprocessing

The data in the dataset may be unstructured or semi-structured encompassing a substantial volume of extraneous information that holds little relevance in the prediction process. Additionally, a sizable dataset necessitates extended training time, and the presence of stop words diminishes prediction accuracy. As a result, computational resources are conserved to enhance the prediction precision aided by text pre-processing. Text pre-processing is vital to enhanced model performance and precise predictions.

**Tokenization**: Continuous text is broken down into symbols, separate words, and elements also called tokens as a result of the tokenization process. The precision and speed of this process significantly impact the effectiveness of the subsequent analysis.

#### 3.3.1 Stop word removal:

This stage is characterized by the elimination of stop words from the Tweets. While stop words enhance sentence readability, they do not contribute meaningful information for text analysis. This stage enhances the classification algorithm’s efficiency.

#### 3.3.2 Short word removal:

Words with fewer than three characters are excluded from the Tweets. This practice of discarding short words in the study serves to bolster the effectiveness of the classifiers.

#### 3.3.3 Case conversion:

Subsequent to the elimination of short words, the tweet manuscript is lower-cased. This step holds significance as the analysis is sensitive to the case. For instance, probabilistic models distinguish between “Bad” and “bad” as distinct words, tallying their occurrences separately. Failing to convert the words to lowercase might hinder the classifier’s efficiency.

#### 3.3.4 Stemming:

Stemming involves stripping off affixes from words, returning them to their base forms. For instance, “plays,” “playing,” and “played” are variations of the word “play” with equivalent meanings. This removal of suffixes aids in diminishing feature intricacy and enhances the classifiers’ learning performance.

#### 3.3.5 Removing @ and symbols:

Stemming phase is followed by stripping @ starting words, as X.com (Formerly Twitter) uses this symbol to denote unique subscriber names. Subsequently, special symbols are also eliminated. However, this study witnessed that even after stripping off the symbols from the tweets, few symbols still remain in the tweets. Consequently, undesirable emoticons (e.g., a heart) are removed in the subsequent step.

In the subsequent step, numerical values are eliminated from the Tweets, because they are of no use in text analysis as they hold no value. Their removal serves to streamline the training complication of prototypes.

### 3.4 ML models for sentiment classification

ML has significantly improved the effectiveness and accuracy of sentiment analysis for data on twitter. There are various sophisticated ML classifiers available for this purpose. The Scikit-learn library in Python offers a wide range of such classifiers. It is an open-source library and has a substantial user community for support. The classifiers employed in the present study are RF, SGD, VC, and SVC.

#### 3.4.1 Support vector classifier.

SVC addresses multi-class issues when expended as a binary classification tool. In the current study, the SVC was employed for both classification tasks whether they are three-class or five-class. This technique proves highly effective in handling nonlinear classification, regression, and outlier detection using kernel tricks [43]. However, it may exhibit reduced accuracy in small datasets since it relies on data cross-validation [28].

#### 3.4.2 Stochastic gradient descent.

SGD algorithm draws inspiration from the convex loss functions of SVC and LR. It excels in handling multi-class classification challenges and is a widely employed classifier. Utilizing the OvA (one-versus-all) approach, SGD combines multiple binary classifiers. Notably, SGD handles just one single example at a time (with a batch size of 1) per iteration. Its simplicity in terms of regression makes it easy to grasp and implement. However, it introduces a significant level of randomness due to its batch selection process. Additionally, achieving accurate results with SGD necessitates precise configuration of hyperparameters. Furthermore, SGD exhibits high sensitivity to feature scaling [23].

#### 3.4.3 Random forest.

RF is a tree classifier. Initially, RF generates numerous decision trees to build a forest based on these random feature sets. Subsequently, the votes from all the decision trees are combined to make predictions on the test data class labels. RF assigns greater importance to the votes from DTs with lower fault rates and vice versa. Low error-rated DTs are focussed in this method and it effectively minimizes the likelihood of incorrect predictions [24].

#### 3.4.4 Voting classifier.

VC enables the fusion of diverse ML classifiers, generating predictions using voting. This classifier is further classified into hard and soft. In hard voting, the outcome is determined by the majority of classifiers. Conversely, soft (weighted) voting involves calculating a weighted percentage for each classifier. It collects the forecasted class probabilities from every record of each model, multiplies them by the respective classifier’s weight, and ultimately averages them to make the final classification assignment [30].

#### 3.4.5 Logistic regression.

LR is the statistical ML algorithm having data classified by considering the extreme values as output variables. It establishes an algorithmic line that effectively separates them. Despite its name, this model is occasionally misconstrued as solely applicable to regression tasks. In reality, it is rigorously deployed for classification tasks being a statistical model. LR stands out as one of the most straightforward ML algorithms, offering high efficiency and low variance. Moreover, It is used as a feature extraction tool. This model can be smoothly using Stochastic Gradient Descent (SGD) with new incoming data [27].

### 3.5 Deep learning models

A great interest has recently been witnessed in employing classifiers based on deep learning techniques. DNNs have the potential to enhance classification accuracy in comparison to conventional classifiers. Thus, this research endeavor seeks to employ a DNN sentimental tweets analysis.

#### 3.5.1 Convolutional neural network.

The CNN is a powerful deep learning architecture widely employed for tasks involving image and text classification. It effectively discerns intricate features associated with the target class during the training process. The CNN comprises multiple layers, including convolutional, pooling, activation, and flatten layers, with the inclusion of dropout layers as well. In the convolutional layer, features are learned from the input data, while the pooling layer reduces the size of these extracted features, resulting in reduced computational complexity. This study utilizes max pooling for experimentation purposes [29,37]. The dropout layer is instrumental in mitigating the risk of overfitting, and the flatten layer serves to transform the data into an array format. The rectified linear unit (ReLU) is employed as the activation function in this study, with a dropout rate set at 0.2.

#### 3.5.2 Long short-term memory.

A variant of RNN, the LSTM model, excels at retaining information over extended periods, aiding in effective back-propagation. LSTMs are composed of memory units known as cells, enabling the retention, modification, and updating of information. The behavior of these cells is regulated by gates that open and close based on incoming signals, determining what information to store, update, or discard. In this study, 300 input-length layers are implanted to prepare each LSTM, utilizing sigmoid and ReLU as stimulation functions. This configuration empowers the model to grasp intricate data patterns [32]. To mitigate complexity and combat overfitting, a random dropout rate of 0.2 applies to neurons. Given the binary classification nature of this study, for LSTM “binary-cross-entropy” is used as a loss function. The optimizer “Adam” is employed to tackle the intricate task of categorizing tweet sentiments. Each LSTM undergoes training for 30 epochs.

#### 3.5.3 Multilayer perceptron.

A DNN is a complex neural network type having hidden layers over two in quantity. DNNs engage in intricate mathematical computations to process data. They find applications in learning word representations for mining text. The words are represented in a neural context referred to as word embedding, which takes the form of a factual-worth vector. This embedding of words allows neural networks to gauge word-match by assessing the in-between distance of two embedding vectors [25,36]. Pre-trained word-embedded neural networks have demonstrated remarkable results across various NLP tasks. Although DNNs can be more challenging to train compared to simple neural networks, they exhibit greater potency in handling complex tasks. DL models adeptly handle computational activities by employing layers arranged in non-linear processing elements’ combinations. This amalgamation of elementary components empowers DNNs to make accurate predictions on novel data. While RNNs and CNNs are extensively utilized for text data and images respectively, in this experiment, we opt for DNNs due to their superior outcomes in a diverse range of NL processing tasks.

Recently, a surge in curiosity regarding the application of neural networks in text mining has been witnessed. The MLP model is employed for acquiring semantic understanding in text classification. Likewise, MLP stands out as the preferred choice for spotting topics within text. Various architectures, encompassing different layer configurations and hyperparameters, are experimented with to attain optimal performance [42]. Furthermore, MLP is instrumental in a diachronic propagation framework, enabling the incorporation of historical context into presently acquired functions using diachronic connections. This method enhances the results of CNNs in text classification. Similarly, the adoption of a DNN architecture for text classification showcases the notable effectiveness of neural networks in handling text data. The results highlight the importance of vector data visualization usage in enhancing the accuracy of classification.

### 3.6 Feature engineering methods

Identifying valuable features within the data is a crucial preliminary stage for effectively training ML classifiers. Extra feature creation using the original dataset improves the efficacy of ML algorithms. This stands as a pivotal factor in elevating its overall performance and improving the accuracy of the learning algorithm. By filtering out irrelevant and redundant data, the desired level of accuracy can be attained. In this context, it’s worth noting that a smaller set of meaningful data outweighs a larger set of irrelevant data. Hence, feature engineering emerges as the procedure of refining meaningful characteristics from unprocessed data, thereby facilitating the algorithmic learning process and augmenting their consistency and efficiency [26,27].

#### 3.6.1 Term frequency-inverse document frequency.

TF-IDF, an important method of feature engineering, is deployed on the unprocessed data to extract valuable information from it. It finds widespread application in fields like sentiment and text analysis. This method weighs each data term in the document and considers inverse document frequency and term frequency. Higher-weighted terms are considered more significant compared to those with lower weights. The weight is determined by the equation provided in Eq (1).

$$W_{i,j}=TF_{t,d}(\frac{N}{D_{t}})$$ (1)

where *TF*_*t*,*d*_ denotes term *t* frequency in a document *d*, *D*_*f*,*t*_ is the document count having the term t and total documents number N in the dataset.

TF-IDF is a widely employed recording method to summarize and retrieve information. TF, or Term Frequency, assesses token frequency and assigns greater significance to more prevalent tokens within a specific document. Conversely, IDF, or Inverse Document Frequency, evaluates tokens that are infrequent in quantity. This means that if proper words are seen in multiple documents, they are believed valuable and significant. TF-IDF is utilized to compute the significance weights of terms, resulting in a weight matrix as the final output. The values within this matrix escalate in correspondence with the TF-IDF count but are counterbalanced by the word’s frequency in the dataset.

#### 3.6.2 Word2Vec.

Word2vec, developed by Google, is a deep learning tool that operates on two models: continuous skip-gram and CBOW (continuous bag-of-words). CBOW focuses on making predictions based on context, whereas skip-gram focuses on one word to predict the surrounding words. Word2vec has demonstrated exceptional performance in tasks like English text classification and clustering. Due to its effectiveness, it was chosen to be integrated into the recommended DL-based model [46].

### 3.7 Proposed approach

**Fig 1:**
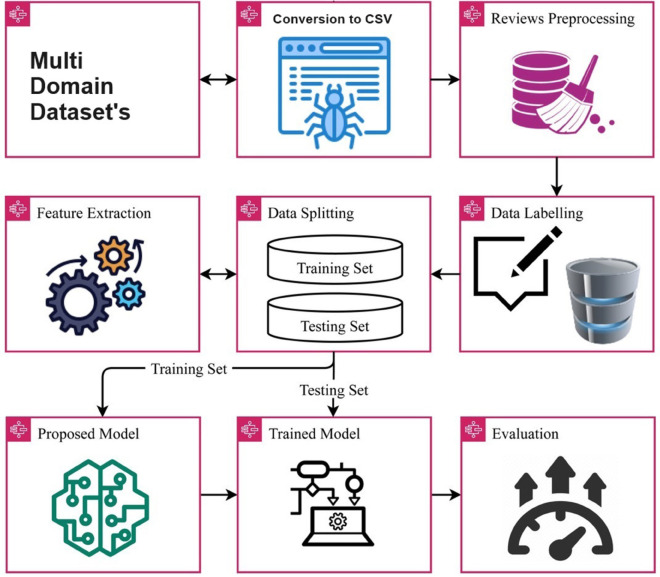
MultiSentiNet architectural diagram.

In this section, we introduce the framework employed here. The depicted context is illustrated in Fig 1. The implementation of deep learning-based classifiers has garnered significant interest in recent years. Deep models hold the potential to enhance the accuracy of conventional classifiers when it comes to classification. Thus, this study focuses on an MLP for the purpose of review classification. The design of the recommended neural network can be observed in Fig 1.

The experiments make use of three benchmark datasets from diverse domains pertaining to text classification. Prior to training, each dataset undergoes five pre-processing steps. The data is then divided into a 70 for training to 30 for testing ratio. Subsequently, the projected approach, which involves combining TF-IDF features with an MLP, is employed for training. The performance of this approach is assessed based on four evaluation metrics: Accuracy, Precision, Recall, and F1-score.

The MLP algorithm is executed using the Keras framework. DNN is used by this algorithm to discern sequences and patterns within the data. Our implemented MLP employs two initiation functions: the rectified linear unit (RELU) and the sigmoid. The RELU function outputs zero for negative values, but positive values are linearly exhibited. Mathematically, relu can be defined as f(z) = max(0, z). The choice of relu as the trigger function is based on its ability to attain high precision across multiple applications. The sigmoid function is rarely favored due to its steady-state at 0. However, in numerous diverse scenarios, relu has proven to produce superior results.

The choice of using the sigmoid function is predominantly suited for difficulties where the aim is to envisage likelihoods as outputs. Since probabilities inherently fall within the range of 0 to 1, sigmoid complements relu in our algorithm. To mitigate overfitting, the “dropout” technique is implemented in our algorithm, as it helps prevent the learning process from becoming overly complex. In our research experiment, we employ 0.2 as a dropout rate in the neural network layers. This dropout technique leads us to utilize 100 training phase epochs, effectively reducing overfitting risk. We apply the optimization algorithm “Adam”, a highly efficient way of training neural networks. We opt for “Adam” as it is commonly employed for RNN in text analysis. “Adam” efficiently determines the most optimal values for the neural network. During training, data is fed to the network in batches of 256 (batch size = 256). We set the input dimension to 2500, reflecting the 2500 features used in our experiment. To compile our neural network model, we specify loss= binary-crossentropy, Adam optimizer, and precision, accuracy, recall, and F1 score evaluation metrics. With these configurations, our algorithm demonstrates exceptional performance, yielding high levels of accuracy. These details in the precise format is shared in Table 4.

**Table 4:** Proposed MLP architecture for sentiment classification.

Layer Type	Output Units	Activation	Additional Configurations
Input_Layer	2500	–	Word2Vec / TF-IDF Features
Dense_Layer 1	1024	ReLU	–
Dropout	–	–	Dropout_rate = 0.2
Dense_Layer 2	512	ReLU	–
Dropout	–	–	Dropout_rate = 0.2
Dense_Layer 3	256	ReLU	–
Dropout	–	–	Dropout_rate = 0.2
Output_Layer	1	Sigmoid	For binary sentiment classification

### 3.8 Evaluation parameters

Four different valuation metrics are deployed to assess the effectiveness of the ML models: precision, F1, accuracy, and recall score. The accuracy score is the representation of the proportion of accurate predictions. Its highest possible value is 1, while the lowest is 0. This is expressed as follows

$$Accuracy=\frac{TP+TN}{TP+TN+FP+FN}$$ (2)

Precision measures the exactness of a classifier. It is a value between 0 and 1, and it is calculated as follows:

$$Precision=\frac{TP}{TP+FP}$$ (3)

Recall reflects the completeness of a classifier. It falls within the range of [0, 1] and is computed as:

$$Recall=\frac{TP}{TP+FN}$$ (4)

The F1 score is a harmonic mean of precision and recall scores. It ranges from 0 to 1, and its calculation is as follows:

$$F1score=2\times \frac{Precision\times Recall}{Precision+Recall}$$ (5)

## 4 Results

In this section, the results of all ML and DL models are compared, and their performance is analyzed in depth. A wide array of experiments was executed, encompassing several ML and DL models, in order to assess their performance using two distinct feature extraction methods: TF-IDF and Word2vec. These experiments were conducted by incorporating the original sentiments. The primary objective behind conducting this diverse set of experiments was to achieve the utmost accuracy in classifying sentiments across datasets originating from different domains.

### 4.1 Results of machine learning models

The chosen classifiers were employed utilizing both Word2vec and TF-IDF features to assess their performance and compatibility with the selected features for each classifier. The training was executed on a 2GB Dell PowerEdge T430 machine, equipped with a graphical processing unit consisting of 2x Intel Xeon 8 Cores 2.4 GHz processors and 32 GB DDR4 RAM. The training process took 3.5 hours on the “Twitter airline database for classification results for 500 epochs.” The experimental outcomes utilizing TF-IDF are presented in Table 5.

**Table 5:** Models’ accuracy using TF-IDF features.

Classifiers	Accuracy		
Dataset’s	D1	D2	D3
LR	79.9%	62.9%	88.0%
RF	75.8%	58.5%	84.4%
SGD	79.2%	62.8%	88.5%
SVC	78.5%	62.0%	88.3%
VC(LR+SGD)	79.2%	63.1%	88.4%

The results depict the highest accuracy of 79.2% for SGD and VC when TF-IDF features are employed for Dataset-1 classification. Likewise, VC and SGD also exhibited superior accuracy compared to the other classifiers for Datasets 2 and 3. While RF showed better results for Dataset 3, its accuracy is relatively low with Datasets 1 and 2. Notably, the overall accuracy is consistently high and comparable for all classifiers with Dataset 3. This is primarily attributed to the fact that Dataset 3 comprises only two classes, leading to enhanced classification performance.

Recall, F1-score, and Precision are computed individually for the neutral, positive, and negative classes using Dataset 1, and the outcomes are detailed in Table 6. The evaluation parameters for performance suggest that SGD outperforms RF, SVC, and VC, for precision, F1-score, and recall. While VC demonstrated a slight variance in performance compared to SGD, RF, and SVC exhibited superior precision but lower recall and F1-score compared to VC and SGD.

**Table 6:** ML models performance using TF-IDF.

Classifiers	Sentiment	Precision	Recall	F1-score
RF	Positive	54%	74%	62%
	Neutral	39%	61%	47%
	Negative	93%	79%	85%
	average	81%	76%	77%
SGD	Positive	64%	74%	69%
	Neutral	49%	65%	56%
	Negative	92%	83%	87%
	average	82%	79%	80%
SVC	Positive	62%	74%	67%
	Neutral	52%	61%	56%
	Negative	91%	83%	87%
	average	80%	78%	79%
VC	Positive	58%	77%	66%
	Neutral	45%	65%	53%
	Negative	94%	85%	86%
	average	82%	78%	79%

The accuracy experienced a slight decrease. TF-IDF exhibited improved performance with ML classifiers due to the dataset’s semantic similarity, enabling the model to achieve superior results. However, on Dataset 2, the performance of SGD saw an improvement with the incorporation of word2vec features. Moreover, there was a minor reduction in the overall classifier performance for Dataset 3 with word2vec as opposed to TF-IDF, as shown in Table 7.

**Table 7:** Models’ accuracy using word2vec features.

Classifiers	Accuracy		
Dataset’s	D1	D2	D3
LR	76.8%	63.1%	86.3%
RF	73.7%	58.6%	84.4%
SGD	78.2%	63.3%	88.5%
SVC	78.0%	63.1%	84.4%
VC(LR+SGD)	78.2%	63.1%	86.5%

Table 8 displays the precision, recall, and F1-score for datasets distinctly, for total included classifiers. This further affirms that SGC and VC consistently outperform others across all three datasets. Notably, evaluation metrics are high for selected classifiers for Dataset 3, as it involves prediction for only two classes. Conversely, the classifiers’ performance declined when Dataset 2, which comprises five prediction classes, was employed. Nevertheless, VC and SGD still outperformed the other classifiers. Additionally, the chosen classifiers were assessed for their performance when utilizing word2vec for specified datasets. The outcome for classifier accuracy can be found in Table 7, revealing a reduction in classification accuracy when incorporating word2vec features.

**Table 8:** ML models performance metrics for all datasets.

	D1			D2			D3		
	Precision	Recall	F1-score	Precision	Recall	F1-score	Precision	Recall	F1-score
LR	79%	80%	79%	59%	63%	58%	88%	88%	88%
RF	54%	74%	62%	49%	59%	47%	85%	84%	84%
SGD	64%	74%	69%	58%	63%	58%	89%	89%	89%
SVC	62%	74%	67%	60%	62%	62%	88%	88%	88%
VC	58%	77%	66%	58%	63%	59%	88%	88%	88%

Table 9 presents the output evaluation metrics for Dataset 1 classifiers when integrated with word2vec. Recall, Precision, and F1-score collectively indicate that the results of ML classifiers witnessed a 0.3 decrease on average when employing word2vec functions for classification. Notably, the VC, SVC, and SGD exhibited similar average performance levels for recall, F1-score, and precision. Conversely, the consequences of RF were significantly diminished when utilizing word2vec functions.

**Table 9:** ML models performance metrics using word2vec.

Classifiers	Sentiment	Precision	Recall	F1-score
RF	Positive	81%	41%	54%
	Neutral	72%	28%	40%
	Negative	73%	98%	84%
	average	74%	74%	70%
SGD	Positive	70%	61%	65%
	Neutral	66%	48%	56%
	Negative	82%	92%	87%
	average	77%	78%	77%
SVC	Positive	72%	62%	67%
	Neutral	66%	48%	56%
	Negative	82%	92%	87%
	average	77%	78%	77%
VC	Positive	76%	58%	65%
	Neutral	67%	49%	56%
	Negative	81%	94%	87%
	average	77%	78%	77%

Likewise, Table 10 provides the recall, precision, and F-1 score for the individually tested dataset. It becomes evident that there is a significant decline for Dataset 2 in comparison to Datasets 1 and 3 when transitioning from TF-IDF to word2vec for feature extraction on the performance grounds. This highlights that the performance of ML classifiers tends to deteriorate when faced with a greater number of classes, as illustrated by Dataset 2, encompassing five classes. Conversely, Dataset 3 metrics remained unchanged between word2vec and TF-IDF, owing to its binary classification nature.

**Table 10:** ML models performance on all datasets using word2vec.

	D1			D2			D3		
	Precision	Recall	F1-score	Precision	Recall	F1-score	Precision	Recall	F1-score
LR	76%	77%	75%	58%	63%	63%	86%	86%	86%
RF	74%	74%	70%	51%	59%	59%	85%	84%	84%
SGD	77%	78%	77%	58%	63%	63%	89%	89%	89%
SVC	77%	78%	77%	58%	63%	63%	88%	88%	88%
VC	77%	78%	77%	58%	63%	63%	87%	87%	87%

### 4.2 Results of deep learning models

The CNN-LSTM and the Deep LSTM mock-ups used word2vec structures for training for a total of 500 epochs. Table 6 displays the outcomes for both testing accuracy and model training including training and testing loss.

Table 11 showcases the results for the accuracy of CNN-LSTM and LSTM prototypes on every dataset. These results indicate that the ensemble model, composed of LSTM and CNN together excels in performance to standalone LSTM classifier. LSTM classifier attained an accuracy of 0.76 as compared to 0.82 of CNN-LSTM on tweets datasets.

**Table 11:** DL models performance on all datasets using word2vec.

	D1				D2				D3			
Classifiers	A	P	R	F1	A	P	R	F1	A	P	R	F1
LSTM	76%	81%	78%	79%	57%	57%	55%	56%	62%	45%	41%	43%
CNN-LSTM	82%	85%	81%	83%	78.1%	74%	85%	79%	92%	91%	92%	91%

The enhanced outcomes of the CNN-LSTM model can be attributed to CNN’s proficiency in handling word embeddings effectively. The enhanced performance is the result of the max-pooling layer that enabled the mining of vital features possible, which served as an input for sequence generation of the LSTM layer. This enabled the CNN-LSTM classifier to surpass in accuracy to other classifiers. Notably, the CNN-LSTM exhibited superior performance across all three datasets utilized for classification.

### 4.3 Results of transformer learning model

BERT is among the foundational transformer-based models that are pre-trained using masked language modeling [45]. BERT has the ability to captures contextual information from both directions and make it most suitable for NLP tasks on benchmark datasets. The BERT consistent performance across all three datasets is shared in Table 12. It achieved 84% accuracy on D1, 76% on D2, and 90% on D3, with corresponding F1 scores of 84%, 74%, and 88%, respectively. Although BERT performed well overall, its results indicate that it may struggle with generalization across different domain datasets, especially D2, which likely contained more domain complexity or imbalanced samples. Still, its ability to deliver high precision and recall on D3 demonstrates robustness in detecting nuanced sentiment or topic variations.

**Table 12:** TL models’ performance on all datasets.

	D1				D2				D3			
Classifiers	A	P	R	F1	A	P	R	F1	A	P	R	F1
BERT	84%	85%	84%	84%	76%	74%	74%	74%	90%	88%	89%	88%
RoBERTa	89%	91%	92%	91%	81%	78%	80%	79%	95%	93%	91%	92%
XLNET	81%	80%	81%	81%	73%	70%	73%	72%	86%	85%	88%	86%

RoBERTa, a robustly optimized version of BERT, further pushes the boundaries of pre-trained language representations by removing the Next Sentence Prediction (NSP) objective and using a larger training corpus and longer sequences. Table 12 results reveals that RoBERTa outperformed both BERT and XLNet on all datasets with the highest scores: 91% F1 on D1, 79% on D2, and an impressive 92% on D3. This significant performance margin highlights RoBERTa’s improved contextual learning and adaptability across varied text domains. Its enhanced pre-training and dynamic masking contribute to better generalization, especially in datasets like D2, where BERT and XLNet lag.

XLNet is an autoregressive model and differs from BERT and RoBERTa by incorporating a permutation-based training objective to capture dependency across the complete dataset. However, its performance in this study was comparatively lower, with F1 scores of 81%, 72%, and 86% for D1, D2, and D3, respectively. Results reveal that although XLNet can capture long-term dependencies, it may not perform as robustly as RoBERTa in classification tasks where fine-grained sentiment or contextual cues are critical. The model’s slightly lower precision and recall in D2 also suggest limitations in domain adaptability or sensitivity to data sparsity.

### 4.4 Results of the proposed MLP model

A diverse variety of classification prototypes is primarily employed to facilitate an inclusive comparison between deep learning and ML algorithms. Given the specific conditions and scenarios considered, it is observed that while the CNN-LSTM exhibits some improvement, MLP, a DL algorithm, ultimately outclasses the rest of the models. Table 13 provides a detailed account of the experimental results obtained using TF-IDF.

**Table 13:** Results of the proposed approach using TF-IDF features.

	D1				D2				D3			
Feature	A	P	R	F1	A	P	R	F1	A	P	R	F1
Word2Vec	86.57%	87.35%	87.68%	87.88%	79.43%	76.97%	79.24%	78.25%	94.09%	92.32%	92.43%	92.39%
TF-IDF	94.75%	95.36%	96.07%	95.87%	84.60%	82.29%	85.17%	83.76%	97.52%	94.73%	96.18%	95.25%

The MLP demonstrates exceptional performance on the test dataset, achieving a remarkable F1 score of 94.75% on Dataset 1 when using TF-IDF features. Across metrics such as accuracy, precision, and recall, the MLP consistently outperforms the other classifiers. In supervised text classification, the MLP proves to be particularly effective. Its adaptability is a key asset, allowing it to learn complex mappings from input to output. This is exemplified by how documented words can be transformed into a single continuous data stream and fed into an MLP, showcasing the model’s flexibility. In our study, the superiority of MLP is attributed to its lack of assumptions regarding the underlying probability density functions or other probabilistic information about the pattern classes, distinguishing it from other probability-based models. This characteristic contributes to MLP’s superior performance on TF-IDF features, even when compared to different datasets used in this study.

### 4.5 LIME

One of the such techniques is Local interpretable model-agnostic explanations (LIME) which is quite beneficial in the field of Explainable AI, particularly in the text classification area. It operates based on building interference of the input data and studying the change that happens in the function’s outcomes. LIME specifically provides local explanations for a single prediction and the figures are understandable by the target user, so they reveal how a precise decision was made. For example, it can explain the top terms or the most significant phrases in the given document that impact the classification made by the developed natural language processing applications LIME. This interpretability however helps the users to have a feel of what the AI models are all about and makes them to be trusted in such sectors as health and finance where the use of AI models is very vital [33].

**Fig 2:**
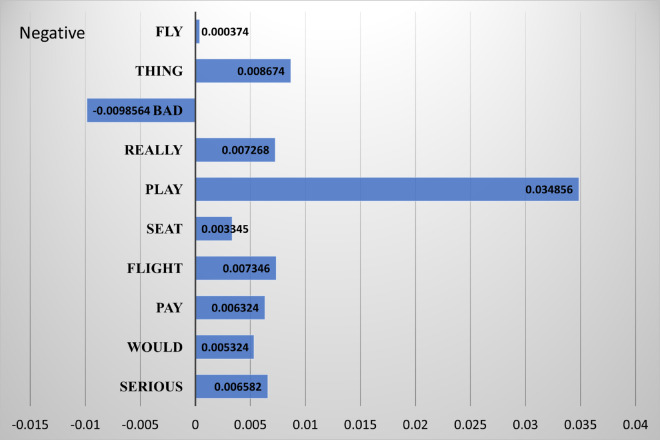
Pictorial representation of misclassified example using LIME explanation.

In this aspect, LIME’s capacity to explain the reasoning of complicated models enriches its uses across various areas and contributes to the appropriate implementation of AI systems. Expounding on the contributions to the fields of transparency and interpretability, it emerges as a beneficial analytical tool for those objective practitioners, and researchers who are trying to understand and verify the results of dynamics of text classification models [34]. Just to express the working of LIME XAI, we have selected 1 example from the US airline dataset that is misclassified. The original label of this tweet is negative but it predicted positive by the model. The misclassification reasoning can be observed from the Fig 2. As it can be seen that most of the words after pre-processing are positive, and only the word “bad” is negative. When model finds commulative confidence score to make this prediction, the support for the prediction of the positive class is more than the negative one. That’s why the model makes this wrong prediction.

Original tweet

@VirginAmerica seriously would pay $30 a flight for seats that didn’t have this playing. it’s really the only bad thing about flying VA

Tweet after preprocessing

serious would pay flight seat play. really bad thing fly

[(‘serious’, 0.006582),

(‘would’, 0.005324),

(‘pay’, 0.006324),

(‘flight’, 0.007346),

(‘seat’, 0.003345),

(‘play’, 0.034856),

(‘really’, 0.007268)

(‘bad’, -0.0098564),

(‘thing’, 0.008674),

(‘fly’, 0.000374)]

### 4.6 Performance comparison of all models

Experiments were conducted using word2vec features to assess the influence of transitioning from TF-IDF to word2vec for feature extraction. The aim was to determine the most appropriate feature extraction method for review classification across diverse domains. The table above displays the performance metrics obtained using word2vec. The results suggest that the performance of the MLP experienced a slight reduction when word2vec features were employed. Table 14 displays the results of all ML and DL models employed in this study.

**Table 14:** Performance comparison of ML and DL models.

	D1				D2				D3			
Model	A	P	R	F1	A	P	R	F1	A	P	R	F1
LR	79%	79%	80%	79%	63%	59%	63%	58%	88%	88%	88%	88%
RF	73.7%	74%	74%	70%	58.6%	51%	59%	48%	84.4%	85%	84%	84%
SGD	78.2%	77%	78%	77%	63.3%	58%	63%	58%	88.5%	89%	89%	89%
SVC	78%	77%	78%	77%	63.1%	58%	63%	58%	84.4%	88%	88%	88%
VC	78.2%	77%	78%	77%	63.1%	58%	63%	58%	86.5%	87%	87%	87%
LSTM	76%	81%	78%	79%	57%	57%	55%	56%	62%	45%	41%	43%
CNN-LSTM	82%	85%	81%	83%	78.1%	74%	85%	79%	92%	91%	92%	91%
Proposed	94.75%	95.36%	96.07%	95.87%	84.60%	82.29%	85.17%	83.76%	97.52%	94.73%	96.18%	95.25%

TF-IDF outperforms word2vec for text classification. Word2vec only represents the frequency of word occurrences in a review and lacks information on word importance, distinguishing between common and rare words. In contrast, TF-IDF encompasses crucial data on the significance of words, which contributes to its superior performance. Additionally, with an expansion in review length, the vocabulary size increases proportionally. This leads to sparsity in word2vec vectors, as larger vector sizes result in numerous zero values. Consequently, the classifiers’ performance is adversely affected by the increased vector size, resulting in a degradation of accuracy when using word2vec features.

The suggested model is compared with the other classifiers employed in this investigation to evaluate its performance. As ML-based classifiers, superior performance is demonstrated by it when employed with TF-IDF features. The table below displays the accuracy of the suggested MLP model in comparison to the ML classifiers. The findings affirm that the recommended model surpasses the performance of the other chosen classifiers across all three datasets utilized in the experiments.

## 5 Conclusions and future work

This study introduces MultiSentiNet, a lightweight but effective multilayer perceptron (MLP) architecture approach with TF-IDF features for multi-domain imbalanced textual data classification. The MultiSentiNet is based on a multilayer perceptron model combined with hand-crafted features for accurate text classification three diverse real-world datasets—US airline sentiments, women’s e-commerce reviews, and hate speech detection—capturing variability in class distribution, domain context, and sentiment polarity. These datasets varied in the number of classes, call sample distribution, and contained customer Tweets. Experimental results demonstrate the superior performance of the MultiSentiNet compared to other machine learning, deep learning, and transformer learning models compared in this research work. The results are further explained using the LIME XAI technique. Furthermore, the results are compared with state-of-the-art approaches to determine the superiority. MultiSentiNet demonstrates cross-domain generalization by maintaining robust performance across different sectors, illustrating its adaptability to heterogeneous textual data. In terms of data bias, I acknowledge the presence of imbalanced class distributions in the tested datasets and specifically designed this approach to remain stable under such constraints. MultiSentiNet offers a lightweight and scalable solution when compared to resource-intensive transformer-based models like BERT and RoBERTa. This makes it practical for real-time or resource-constrained environments while retaining competitive accuracy. Future directions involve exploring hybrid word embedding feature engineering techniques to enhance feature learning and extraction. Furthermore, fusion of contextual and sentiment level POS tagged features can also be utilized for better results.
